# Electro-Magnetism in a Case of Poisoning, with Suggestions for Its Application to Still-born Children, and Some Forms of Disease

**Published:** 1843-05

**Authors:** Thomas S. Page

**Affiliations:** Valparaiso


*Electro-Magnetism in a Case of Poisoning, with Suggestions for its application to still-born Children, and some forms of Disease.*—By Thomas S. Page, M.D., of Valparaiso.—A. T., an Englishman, the subject of this communication, aged twenty-two years, and of robust frame, is a clerk in one of the most respectable commercial houses in Valparaiso. He had a slight gleet, for which he prescribed himself pulverised cubebs of doses of half an ounce, night and morning, and experienced from them neither good nor bad effects. On the night of the 16th of March, 1842, he went to an apothecary's shop and asked for cubebs. Not having confidence in the lad in attendance, he requested permission to examine the label on the bottle, and read thereon "Pulv. Cubeb." He then ordered an ounce, divided into two parts, and with these returned home at midnight. He immediately took one of the powders, placed himself in bed, and, as was his custom, took up a book to read, but, as he expresses it, had not read two lines before he felt a dizziness and inclination to sleep. I accidentally discovered him the following morning about twelve o'clock with these symptoms: face red and swollen; lips dark-purple; mouth containing a viscid frothy saliva; tongue has a dry and chapped appearance in the centre, and the teeth are slightly coated with a brown sordes; veins of the forehead and temples turgid; eyes rolled upwards, injected, and their pupils contracted to a point; skin moderately warm and moist, with clammy perspiration; feet cool; pulse very slow, moderately full, and dispersed by the least pressure; respiration very slow, short, and gasping. By agitating him violently he was aroused for a moment, uttered some incoherent expressions, and sank back into comatose sleep.

These were the symptoms when I first saw him. Dr. Houston of the Royal Navy, now practising in this place, and Dr. Barrabino, of the U. S. Navy, then attached to the U. S. schooner, *Shark*, came to my assistance. We administered the sulphate of zinc as an emetic, and hot mustard and water to arouse the sensibilities of the stomach to its impression. Large draughts of this, and titillation of the fauces, produced vomiting, and a small quantity of the powder apparently was brought up. The stomach pump was at hand, but as vomiting was readily provoked, it was not used. The patient was made to sit on the edge of the bed with his feet hanging in a tub of almost boiling water strongly charged with mustard. One cup was applied to each temple, and about two ounces of blood abstracted. Large sinapisms were spread over the chest and stomach and inner parts of the thighs. A very strong liniment of ammonia, cantharides, and turpentine, was applied to the whole length of the spinal column, until the skin became very red and inflamed. When the stomach seemed to be cleared of all traces of the poison, the mustard draught was suspended, and a large quantity of olive, with castor oil, administered, but only a part remained. The patient now appeared to be sinking. The surface was cold and covered with a damp sweat; the face was pallid, with a purplish tinge; the jaw and eyelids were fallen, when the patient, by powerful sternutatories and severe blows on the face and shoulders with the open hand, was with difficulty made to rise. Ammonia and brandy and water were now given, with light broths, and an injection composed of turpentine and ammonia. This produced a slight discharge from the bowels. The stimulating liniment already mentioned was repeated to the spine and over the surface of the body. The pulse was hardly perceptible at the wrists, if, at times, it was at all to be felt. The stimulants were continued.

It was now 3 p. m. There were no signs of reaction, and the features wore the aspect of death. Under these discouraging circumstances, and when every effort seemed vainly expended, we now determined to dress the patient and supported by two strong assistants, to take him from his room, continue the stimulants and light broths, and endeavor to walk him in the cool air. At first he made feeble but unsuccessful efforts to direct the movements of his legs, but at length could not be aroused, made no effort to stand, and sank almost lifeless into the arms of his assistants.* He was carried to his room and placed in a slightly reclining posture on his bed. His breathing was now short and hurried; his mouth wide extended and jaw fallen; nothing seemed capable of arousing him; the exhaustion was extreme; the pulse could be felt feebly at the wrist, maintained there probably by the agitation which he had just undergone. Dr. Houstoun had left at a short time previous, Dr. Barrabino remained with me.

It was now 4 p. m., and worn out with fruitless efforts we desisted entirely from farther exertion. At this conjuncture I thought of my electro-magnetic battery, and proposed its application to bring about reaction, for I felt we were justified in such desponding circumstances to make it a matter of experiment. Cerebral congestion was urged as an objection, but admitted not to be sufficient, in such a desperate case, to set aside the experiment. It was immediately tried, and with the happiest results. With an assistant rapidly rotating the wheel, I applied the balls at first to each side of the neck, and ran them down behind the clavicles. The arms and body now moved convulsively, but the patient lay as unconscious as before. I now passed one ball over the region of the heart, and the other to a corresponding point on the right side. In an instant his eyes opened widely, and with a ghastly expression of countenance; his head and body were thrown convulsively towards me, and he groaned. He now sank back into his reclining posture, and he was again asleep. The balls were re-applied in the same situation with similar results—a third and fourth time, and he cried, "No more." Reaction was now positively established; the heart had received a

* Broths and stimulants were poured into his mouth, but he could no longer swallow them.

strong impulse; the pulse was becoming rapidly developed, and the whole surface warm.

We now determined to desist, and watching him attentively, allow him to remain quiet for an hour. Reaction continued satisfactorily, and when the hour had expired he could be awaked by shaking his body and calling loudly his name. There was no further occasion for the battery. He was aroused at intervals; and at eleven o'clock in the evening was sufficiently awake to relate where he had got the medicine the preceding night, but was still drowsy, and when not disturbed inclined to sleep. Thus he passed the night, and on the following morning was pretty well. He then told me that he had heard many things the preceding day that were said by persons about him, but he neither felt the power to open his eyes nor move his tongue to speak, although up to 3 p. m., when powerfully agitated and spoken to, he would reply in short and sometimes broken sentences, and occasionally correctly. He further says that the last thing he has any recollection of was my remark. whilst they were attempting to walk in the corridor, that nothing more could be done but to make the experiment. From that time all was blank to him, until, as he expressed it, "he felt as if a gun had been fired off within him, which thrilled through and shook him to the very extremities." This was the application and effect of the electro-magnetic battery.

I have said that cerebral congestion was thought at the time to make the application of the battery in such cases objectionable. The result proved the incorrectness of this opinion, and sustains this argument in favor of the practice adopted, viz:—By observing the phenomena of diseases a relation may be remarked between some of them in their earlier stages, whose terminations and consequences are quite dissimilar; apoplexy and epilepsy furnish an example. In both there is great cerebral congestion. The former generally terminates in effusion, and paralysis is the consequence; the latter terminates in spasms, and the patient returns to his usual health. Therefore it would seem that the muscular spasms equalise the circulation, and thus unload the brain; or, if we might suppose epilepsy to depend upon a determination, if I may use the expression, of the nervous principle to the nervous centres, the latter are relieved by throwing it off upon the nervous extremities, occasioning thereby spasm. Viewing these vital actions as the efforts of nature to relieve organs from the effects of undue accumulation, and restore the equilibrium in the nervous and vascular systems, it appears probable that severe narcotism of the nervous centres may be diffused and shaken off by the revulsive action of the battery on the nervous branches, and that the consequent developments of vital action would give an impulse to the general circulation which might relieve the cerebral congestion.

A question might arise as to the power of the medicine taken to destroy life. On this point the melancholy death of Mr. C., a French gentleman, late of this place, who took the same medicine and in the same quantity but a few weeks previous, affords convincing testimony. In illustration of my subject I asked Dr. Cazentree, a French practitioner of this place, and the physician of Mr. C., for an account of the circumstances attending his death, and the autopsy, which he very politely communicated as follows:—

"Mr. C. was afflicted for some time with a gonorrhœa, for which, without medical advice, he took balsam copaiva, even in large doses, but to no effect. He was now attacked with orchitis, and for the first time came to consult me. After eight days of constant attendance the swelling which existed in the left testicle disappeared, but the blenorrhagia returned with more force. Vexed with this he again wished to take remedies which might relieve him at once of this afflicting disease. I recommended the use of the cubebs, which, taken for nine days, and gradually augmented to two drachms three times a day, had almost completely taken away this obstinate affection, when, on the 13th of February, not having any of the remedy left, he sent the same recipe to the nearest apothecary's shop.* At ten o'clock at night he retired to his bedchamber well and cheerful. Without consulting any one, on lying down he took half an ounce of cubebs. No noise whatever was heard during the night, and at seven o'clock the following morning, when they entered his bed-chamber, they found him in a state of insensibility. Half an hour after I was with him, assisted by Dr. Veillon, and we found him in the following condition:—

"The body is in a state of supination; all the senses are extinguished, without hearing, speech, or movement; the eyelids are fallen, and when raised the eyes look cloudy and fixed; the pupils are dilated; extremities flexible; they obey the hand which raises them, and fall like an inert body; heat nat-

* He had previously got the cubebs at another shop, but on this occasion purchased it at the same shop where my patient subsequently procured his.

ural and equally diffused; face red; there are colored, blackish spots on various parts of the body, but principally on the back; when the body is moved a species of strong râle is heard in the bronchia; pulse slow, feeble, and very irregular; respiration hardly perceptible. Not knowing to what to attribute a state so suddenly produced and so grave, and recognising by the symptoms the appearance of asphyxia, and thinking he might have taken too strong a dose of the medicine, Dr. Veillon and myself proceeded in consequence to extract from the stomach the cubeb that it might yet contain, and to cause reaction by the most powerful excitants. But all was in vain, and at twelve (noon) life was completely extinct. Nothing now remained but to make the autopsy, which we did the following morning at seven o'clock. All the cavities and organs were examined with the greatest care.

"*Exterior.*—The face is pallid, the integuments livid, principally behind, and the corpse rigid.

"*Abdomen.*—The stomach with no trace of inflammation, contains a tumblerful of liquid, in which is observed a little of the powdered cubebs, mixed with some aliment, which is almost digested; the intestines are sound and healthy; the bladder is full of crystalline urine; the liver, spleen, and kidneys are full of black and fluid blood.

"*Thorax.*—The lungs are excessively engorged with blood, and when cut into with the knife this flows with a great abundance of froth from the bronchia; the left side of the heart is entirely empty, the right full of blood; the aorta and all the arterial system is entirely empty; the venous system of the thorax and abdomen, as well as the pulmonary artery, the vena cava and portæ, are full of black and fluid blood, which flows abundantly as soon as the vessels are divided.

"*Head.*—The veins of the brain are also congested, but the congestion of this organ is not as great as that observed in the thoracic and abdominal viscera. I repeat, that in no part was there red or coagulated blood found, but always black and fluid, and filling all that appertained to the venous system."

Before closing this subject, I would beg leave to add my impression that electro-magnetism will not only be found a most useful agent in cases like the above, but in some forms of disease, particularly those of a highly congestive character, where oppression of the organs and the nervous system prevent reaction and speedily destroy life. I need not occupy space in adducing cases illustrative of my meaning. In practice I think we frequently see cases where death seems to be caused by an *obstruction* of the functions or organic movements which support life, more than by an *exhaustion* of the organic functions or of life itself. And in such cases electro-magnetism might communicate an impulse which would renew those sympathetic actions between the organs (if no positive lesion exist in any of them) upon which the continuance of life depends.

In all cases of asphyxia, electro-magnetism must be useful; and I am strongly impressed with the belief that it might be applied in very many instances to still-born children with the happiest effects; for this purpose an instrument might be used of a very portable form,—that used by me in the case related consists of a large *horse-shoe magnet*, mounted upon a stand, in a vertical position, with an *armature*, fixed upon an axis between the poles, so as to revolve in front by means of a wheel. The *armature* consists of two cylindrical bars of soft iron, connected by a cross-bar, and in the *centre* of the bar is an insulated ferule; as near the end of the bar as possible is fastened the "*breakpiece.*" Around each of the cylindrical bars is wound *two thousand two hundred and fifty feet of wire*, covered with cotton thread, to prevent the current of electricity from passing from one wire to the other; the end of *one* of the coils is connected with the "*breakpiece*," and the other with the *ferule*. From one of the pillars, which are in *front* of the armature, the *springs* are made to act on the ferule and breakpiece. From the other pillar the spring connects with the *centre;* the *handles* are fastened by the *set screws* in the base of the pillars. There are four set screws in the back of the upright block of wood to set out the magnet, so as to make the armature revolve as close as possible to the magnet. The shock is communicated on ordinary occasions by grasping large brass handles which connect with the instrument by short coils of wire, which are painted red. To apply it to different parts of the body long wires, covered with cotton-thread, and terminating in brass balls, are used. Two glass cylinders enclose the wire near the balls, for the operator to hold by while administering the shock.

The above article, which we copy from the London Lancet, is also published in the American Journal of the Medical Sciences, for April, just received. Dr. J. Warrington, through whom it was transmitted to the latter Journal, received at the same time two parcels, one containing the half-ounce of powder corresponding to that taken by the patient; the other a substance purporting to be cubebs, and bought at the same shop. These were given to Mr. Procter, Jr., for examination; he publishes an analysis of them in the American Journal, from which we copy so much as will show the nature of the deleterious substance.

The substance submitted for investigation consisted of two specimens; one labeled "sample of the powder corresponding to that which the patient took;" the other, "sample of the powder purchased by Mr. Peel whilst the patient was under the influence of the poison, who at my suggestion, went to the same shop and asked for an ounce of pulv. cubebs." They were severally enveloped in double papers and sealed; the first weighing about 100 grains, the second 200 grains; and their sensible properties accorded in all particulars.

The powder was dry, finer than cubebs ordinarily occur, and without the moist appearance usual to it. Its color was a uniform brown, its odour less aromatic and agreeable than that of good cubebs, and its taste pungent, though accompanied by a bitterness not found in the genuine drug.

In the paper accompanying the specimens, Dr. Page does not express an opinion as to the nature of the poisonous agent associated with the cubebs. A consideration of his remarks on the symptoms produced by the powder, suggested the idea that opium mixed with cubebs might produce all the effects there detailed. The sensible properties of the substance sustained this impression, and the addition of tannic acid and a persalt of iron to its decoction giving indications favorable to the presence of morphia and meconic acid. was considered sufficiently encouraging to cause a pursuit of the investigation with a view to the presence of opium.

The following is a summary of the substances isolated.

| | In 100 parts. |
|---|---|
| Cubebin or piperin, | 2.25 |
| Morphia, | 2.50 |
| Meconic acid, | 1.25 |
| Narcotine, | Proportions not ascertained. |
| Narceia, | |
| Volatile oil and resin, | |
| Extractive and gum, | |
| Chloride of sodium, | |
| Lignin, | |

As the object of this analysis is to ascertain the nature and properties of the deleterious agent associated with the cubebs, only those parts of the investigation have been given in detail, which, by proving the presence of morphia and meconic acid in the powder, render the existence of opium in it evident. That other principles, as codeia, meconin, etc., were also contained in it, there cannot be a doubt, but search for them would be as hopeless as it is useless. In conclusion, it may be observed that the quantities of morphia and meconic acid indicate the presence of about 30 per cent. of opium in the powder, equal to 75 grains in the dose taken by the patient, and doubtless sufficient to have occasioned death.

